# Factors affecting larval tick feeding success: host, density and time

**DOI:** 10.1186/s13071-015-0955-6

**Published:** 2015-06-24

**Authors:** Cami R. Jones, Jesse L. Brunner, Glen A. Scoles, Jeb P. Owen

**Affiliations:** Department of Entomology, Washington State University, Pullman, WA USA; School of Biological Sciences, Washington State University, Pullman, WA USA; Animal Disease Unit, USDA-ARS, Washington State University, Pullman, WA USA

**Keywords:** Parasite development, Host defense, Parasite density, Host-parasite interaction, *Dermacentor andersoni*, *Peromyscus maniculatus*

## Abstract

**Background:**

Ectoparasites rely on blood-feeding to sustain activity, support development and produce offspring. Blood-feeding is also a route for transmission of diverse vector-borne pathogens. The likelihood of successfully feeding is thus an important aspect of ectoparasite population dynamics and pathogen transmission. Factors that affect blood-feeding include ectoparasite density, host defenses, and ages of the host and ectoparasite. How these factors interact to affect feeding success is not well understood.

**Methods:**

We monitored blood-feeding success of larval Rocky Mountain wood ticks (RMWTs; *Dermacentor andersoni*) on deer mice (*Peromyscus maniculatus*) in several experiments to determine how tick density, host defense, and ages of mice and ticks interact to influence feeding success. In the first experiment, tick-naive deer mice were infested with one of several densities of RMWT larvae, while a second cohort of mice were infested with 50 larvae each. Two weeks after ticks dropped off, mice in the first cohort were re-exposed to 50 larvae each and mice in the second cohort were re-exposed to varying densities of larvae. In the second experiment mice of different ages (45–374 days old) were exposed to 50 larvae each. Two weeks later mice were re-exposed to 50 larvae each. We combined data from these and several similar experiments to test the generality of the patterns we observed. Lastly, we tested whether tick feeding success was consistent on individual mice that were challenged on four occasions.

**Results:**

Mice acquired resistance such that feeding success declined dramatically from the first to the second infestation. Feeding success also declined with tick density and tick age. Mice, however, became more permissive with age. The sizes of these effects were similar and additive. Surprisingly, over successive infestations the relative resistance among mice changed among hosts within a cohort.

**Conclusions:**

We predict that larval blood-feeding success, and thus development to the nymph stage, will change due to variation in tick age and density, as well as the age and history of the host. Incorporating these biotic factors into modeling of tick population dynamics may improve predictions of tick-borne pathogen transmission.

**Electronic supplementary material:**

The online version of this article (doi:10.1186/s13071-015-0955-6) contains supplementary material, which is available to authorized users.

## Background

Transmission of vector-borne pathogens is strongly affected by arthropod abundance; higher vector densities increase biting rates, which elevates the risk of pathogen transmission [1]. Blood-feeding arthropods (vectors) rely on the blood meal to support development and reproduction [2–4]. To date, most studies of vector blood-feeding have focused on either (i) differences in host-vector contact due to host movement in space [5–7] or (ii) differences in host resistance to vectors such as grooming behavior [7, 8] or immune responses [9–11]. Relatively little is known about the intrinsic properties of hosts and vectors that might affect the probability that an ectoparasite will successfully feed, develop and reproduce.

Ectoparasite burdens on wild animals vary with many properties of the host including sex, age, body size, and physical condition [12–15]. Among these, differences in parasitism between host sexes is perhaps the best documented. For example, Ixodid ticks are generally found more often and at higher densities on male hosts (mice, voles, and other small mammals) [6, 12, 15–17]. These differences in tick burden may reflect variation in exposure to host-seeking ticks. Male rodents often have a larger home range compared to females [5, 11] and may encounter more questing ticks. Alternatively, males may differentially exhibit behavioral traits after contact with parasites. For example, grooming behavior removes ectoparasites from the host [8] and male impala have been observed to groom less than females, resulting in higher tick burdens [18]. Grooming behavior of hosts has also been studied relative to the host’s body size and age [8, 19, 20]. For example, Mooring and Hart found that the rate of tick removal via self-grooming was three times greater in newborn impala compared to adult mothers [20].

Another key host trait is immunological defense, which affects how susceptible (permissive) the host is to ectoparasites [21, 22]. Host immune defense can be affected by past infection, age, diet, stress, endocrine factors and genetics [21–26]. In particular, past infection can strongly shape immunity by triggering adaptive responses that increase the strength and specificity of immune defense [27]. Hosts with prior exposure to an ectoparasite are often more resistant than naïve hosts. For example, deer mice (*Peromyscus maniculatus*) repeatedly infested with the tick *Dermacentor variabilis* produced inflammation at the feeding site that decreased the amount of blood consumed, and decreased survival of the ticks after engorgement [10]. In a study with *Ixodes sp*. ticks infesting voles (*Clethrionomys glareolus*) and wood mice (*Apodemus sylvaticus*), Hughes and Randolph observed decreased blood-feeding over four sequential infestations and the authors also observed greater defense when hosts were injected with tick salivary proteins and subsequently infested with ticks [11]. These data suggest that ectoparasite infestations will alter host permissiveness to blood-feeding over time. Aging in hosts can also affect susceptibility to infection [28, 29]. For example, when old mice were infected with *Salmonella typhimurium* they were more susceptible to infection and had greater colonization of the bacteria throughout the body compared to young mice [29]. Old mice also had impaired function of the cytokine responses that are involved with defending against *S. typhimurium* [29]. This suggests that immune function may change as hosts age and alter how they respond to infection.

As with the host, the properties of the ectoparasites can vary in ways that affect blood-feeding success. One example is the ectoparasite burden (individuals per host). As hosts encounter ectoparasites in the environment over time, or in different locations, the number of ectoparasites on the host can change. Interestingly, the effect of ectoparasite burden on blood-feeding success is mixed. For example, feeding success declined at higher densities of *Ixodes trianguliceps* larvae on common laboratory mice, whereas no change in feeding success was observed at higher tick densities when fed on their natural rodent host, *A. sylvaticus* [30]. Levin and Fish observed significantly fewer fed larval *I. scapularis* at higher densities when feeding on their natural host, white-footed mice [31], but Hazler and Ostfeld observed no effect of tick density on blood-feeding in the same system [32]. These various studies illustrate that feeding success may change depending on the number of attached ectoparasites. It is also possible the ectoparasites that attach vary in condition, such as size and age. Some studies have implicated an effect of tick age on feeding success. For example, Hazler and Ostfeld observed a significant decrease in feeding success of *I. scapularis* larvae on white-footed mice over a two-month period, which they attributed to aging of the larvae [32]. Although several factors are known to influence the feeding success of ticks and other ectoparasites, most studies only address single factors [8, 12, 14]. It remains unclear whether host and tick characteristics are equally important, or if they interact to affect feeding success. In addition, field studies suggest that intrinsic characteristics of a host produce a consistent level of susceptibility relative to other hosts in the population [33, 34]. However, it remains unclear if hosts maintain consistently high or low ectoparasite burdens due to differences in tick contact (e.g., foraging range) versus factors after contact (e.g., defense).

Here we ask how blood-feeding success changes with the age and prior exposure of the host, and with the ectoparasite’s age and density. We studied a natural host-ectoparasite association between the Rocky Mountain wood tick (RMWT; *Dermacentor andersoni*) and deer mice (*P. maniculatus*). The RMWT is endemic to the western United States and is responsible for transmission of several pathogens that impact human and livestock health [35]. Similar to *Ixodes sp*., the RMWT is a 3-host tick that utilizes small rodents as hosts during the larval stage [35, 36]. Using this naturally occurring host-ectoparasite association we conducted controlled infestations of deer mice with larval RMWTs and measured tick feeding success as a function of prior infestation, tick density, mouse age and tick age. We discuss the results of the experiments in the context of tick density, population dynamics and pathogen transmission.

## Methods

### Mice

Deer mice (*P. maniculatus*) were originally obtained from the Peromyscus Genetic Stock Center (University of South Carolina) to start a laboratory colony. Mice were kept in a climate controlled room at c.a. 21 °C, a relative humidity of c.a. 30 %, and in a 16 h light: 8 h dark cycle. Mice were housed single sex with 2 – 6 individuals per cage until used in experiments when mice were held in cages individually. No mice in breeding pairs were used in experiments. Mouse ages ranged from 2 to 16 months and the colony had an approximate 1:1 sex ratio. All mice were fed *ad libitum* using a breeder-type diet of laboratory rodent chow (Harlan Laboratories). All animal use was approved through the Institutional Animal Care and Use Committees (IACUC) of Washington State University and the University of Idaho.

### Ticks

Ticks used in these experiments were from a colony of Rocky Mountain wood ticks (*D. andersoni*) derived from field caught adults in Reynolds Creek, Idaho. Adult ticks were fed on Holstein calves to produce eggs [37]. Hatched larvae used in the experiments ranged in ages from 19 to 118 days post-hatch. The larvae ranged from 0 to 4 generations removed from field-collected ticks. Only one subset of larvae used in each of the following experiments was four generations removed from the field population. Prior to use in experiments the larvae were kept in glass vials in humidity controlled chambers. Humidity was maintained using a saturated salt solution of potassium nitrate to keep larvae at c.a. 75 % relative humidity [38]. Humidity chambers were then kept in an incubator at 15 °C and in a light cycle of 12 h light: 12 h dark. The light cycle was similar to what larvae experience in the field when host seeking during the spring and summer months where the day length is approximately 11 – 15 h long (sunrise to sunset from March to June PST) [39].

### Infestation procedure

Fixed numbers of RMWT larvae were brushed into a 9.83 cm long, 5.08 cm diameter cardboard tube (Bio-Serv). Each tube was placed in a solid, plastic mouse cage measuring 29.21 cm L × 21.27 cm W × 15.88 cm H with a small handful of bedding material (TEK-Fresh Laboratory Animal Bedding, Harlan Laboratories). An individual mouse was placed in each cage containing ticks and the mouse encountered the ticks (became infested) when it used the tube as refuge over a 48 h period. Each infested mouse was then transferred to a wire-bottom cage measuring 29.85 cm L × 20.96 cm W × 15.24 cm H along with the cardboard tube for environmental enrichment. The tube was checked for any unattached larvae before placing it in the wire-bottom cage with the mouse. The plastic cage was checked for any unattached larvae before discarding the bedding. No larvae were found in either the tube or the cage at the time of mouse transfer for any of the infestations. Once the mouse had been transferred, the wire-bottom cage was suspended over a pan of water. Replete larvae that dropped from the mouse were collected from the water-pan each day over a 5 days period. Mice were checked for any larvae still attached on day 5 during the transfer back to the solid cage, or before euthanasia. In all cases, no larvae were attached after this period for any of the experiments conducted. Feeding success was determined from the total number of replete larvae recovered from a given mouse. These replete larvae represented ticks that were able to successfully get on the mouse, attach to the mouse, fully engorge and drop into the water-pan. Recovered ticks were transferred to an incubator (75%RH; 25 °C; 12 h light: 12 h dark) and molting success was determined by counting the number of ticks that successfully completed the molting process.

### Experiment 1: Variation in dose of first infestation or second infestation

Tick-naïve mice, *n* = 20, were divided into five groups of four mice each. All mice were weighed (±0.01 g) prior to allocation into treatment groups and host weights were distributed evenly between the tick treatment groups to avoid confounding effects of body size. Each group was infested with a different dose of RMWT larvae; a mouse within a group encountered 10, 25, 50, 100 or 250 larvae. Those densities represent a natural range of tick burdens with 250 being at the upper end of the distribution [40]. There were two male and two female mice in all groups except for the group that received 10 larvae each that used three male mice and one female mouse. Larvae were recovered from each mouse over a 5 days period and held until molted (see above). After the 5 d drop period there was a 2 w resting period before a second infestation [10, 11, 41]. After this period, all mice were exposed to 50 larvae each. All mice used in this cohort (cohort 1) were c.a. 140 days old at the start of first infestation and c.a. 161 days old at the start of the second infestation. Ticks used at the start of the first infestation were c.a. 19 days old and c.a. 40 days old at the start of the second infestation.

A second cohort (cohort 2) consisted of 20 tick-naïve mice infested with 50 RMWT larvae each using the methods described above. Larvae were recovered from each mouse and held until molted. After the first infestation, mice rested for 2 w before a second infestation [10, 11, 41]. Mice were weighed (±0.01 g) prior to their second infestation and were distributed evenly into five groups (4 mice/group). Mice in a group were infested with one of the following densities of larvae/mouse: 10, 25, 50, 100 or 250. There were two male and two female mice in each of the five groups. Engorged ticks were recovered from each mouse and held until molted. Feeding success and molting success were determined in the same manner as for cohort 1. All mice in cohort 2 were c.a. 57 days old at the start of the first infestation and c.a. 78 days old at the beginning of the second infestation. Ticks used for this cohort were c.a. 97 days old at the beginning of the first infestation and c.a. 118 days old at the start of the second infestation.

### Experiment 2: Variation of mouse age

Mice, *n* = 18, ranging in age from 48 days old to 374 days old were used to test the effect of host age on development of resistance. There were five male and 13 female mice used in this experiment based on what was available in the colony. All mice were infested with 50 RMWT larvae following the procedure described above. All blood-fed larvae were recovered, stored and held until molted into nymphs. After a 2 w resting period all mice were infested with 50 RMWT larvae for a second time. Mice at the start of the second infestation ranged in age from 62 days old to 388 days old. Engorged larvae were recovered and held until molted. Weights (±0.01 g) for all mice were obtained prior to each infestation period. Ticks used in the first infestation were c.a. 58 days old at the start, and ticks used in the second infestation were c.a. 72 days old at the start.

### Post-hoc data

In addition to the mice used in the two experiments described above, 38 mice (13 males and 25 females) from the colony were infested with ticks two times following the methods above in preparation for other experiments not reported here (see Additional file 1). During those two infestations we controlled tick density, recorded ages of mice and ticks, and measured feeding success. Combining the data from eight controlled experiments, including the two experiments described above, we had a total of 192 observations of feeding success from 96 individual mice. These data were used in a larger, *post-hoc* analysis of age, density and prior infestation. Twelve out of the 38 mice described in this section were infested with ticks a total of four times following the methods described above (see Additional file 2). The first 2 infestations of those 12 mice were included in the combined dataset used in the *post-hoc* analysis. We evaluated the relative ranks of resistance among those 12 mice at each of the four infestations to test the consistency in feeding success on mice over multiple infestations.

### Statistics

The binomial feeding success data (i.e., fed or not fed) was analyzed with a logistic generalized linear mixed model using the glmer () function in the lme4 package [42, 43] in R, version 3.0.2 [44]. We included both random intercepts for individual mice and random slopes describing how feeding success changed from the first to the second infestation. When multiple experiments were analyzed together, we included a random intercept for experiment. The number of ticks a mouse was exposed to was log_10_-transformed, and the ages of mice and ticks were centered on the mean age before analysis. Fixed effects included ages of the tick and mouse, sex of the mouse and density of the ticks. We report the Wald z-statistics and associated P-values for the main effects, which are asymptotic approximations (see: http://glmm.wikidot.com/faq). This framework was used to determine the effect size of prior host exposure, tick density, host age and tick age on blood-feeding success of larvae.

## Results

### Experiment 1: Variation in dose of first infestation or second infestation

**Fig. 1:**
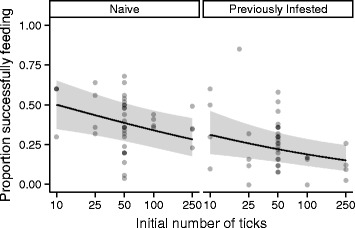
Variation in dose of first infestation or second infestation. The proportion of ticks that successfully fed on naïve (left) or previously infested (right) deer mice as a function of the number of ticks to which they were exposed in experiment 1 (cohort 1 & 2). Points are semi-transparent to show over-plotting. Lines and gray regions are predicted values and 95 % confidence intervals from the generalized linear mixed model without accounting for the random effects

An average of 32.1 % of ticks successfully blood fed when the density of ticks varied in the first exposure (cohort 1) or the second exposure (cohort 2). The odds of successfully feeding declined dramatically when the tick density increased for each cohort. Feeding success decreased by approximately half (e^βdose^ = e^−0.667^ = 0.513; *z* = −2.691, *P* = 0.007) with every 10-fold increase in the number of ticks a mouse encountered (Fig. 1). The surface area of a mouse, estimated as mass^2/3^, did not significantly influence feeding success (*z* = −0.807, *P* = 0.419). Thus we excluded host mass from this and subsequent analyses. There was a strong, negative effect of prior infestation on feeding success (*z* = −5.699, *P* < 0.001). Tick larvae were roughly half as likely to successfully feed on previously infested mice compared to naïve mice (e^βExposed^ = e^−0.796^ = 0.451; Fig. 1). There was no evidence of an interaction between second infestation tick densities and the previous infestation (*z* = −0.515, *P* = 0.607) as would be expected if previously infested animals responded more aggressively (e.g., greater immunity or grooming) to increasing densities of ticks. Similarly, feeding success during second infestations was not affected by the number of ticks in the primary infestations (*z* = −0.675, *P* = 0.499 in a logistic model without random effects).

The magnitudes of the random effects of mouse identity were similar to the fixed effects of tick density and prior infestation. The standard deviation of the intercept (i.e., mouse-to-mouse variation in feeding success for naïve mice) and slope (mouse-to-mouse variation in the change of feeding success when mice were exposed a second time) were 0.604 and 0.714, respectively, on the logit scale. The standard deviation of the random effect of experiment was roughly half as large (0.334) as the effect of mouse identity.

### Experiment 2: Variation of mouse age

**Fig. 2:**
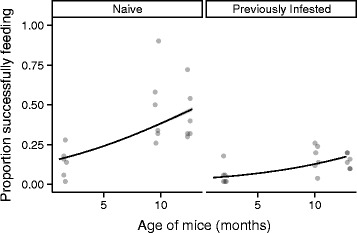
Variation in mouse age. The proportion of ticks that successfully fed on naïve (left) or previously infested (right) deer mice as a function of host age in experiment 2. Points are semi-transparent to show over-plotting. Lines and small, gray regions are predicted values and 95 % confidence intervals, respectively, from the generalized linear mixed model without accounting for the random effects

An overall average of 23.6 % of RMWT larvae successfully fed when the ages of the mice were varied. Ticks were more likely to feed successfully on older mice than on younger mice (*z* = 27.845, *P* < 0.001, Fig. 2). For every month of age increase, the odds of successfully blood-feeding increased 1.14-fold (= e^βAge^ = e^0.134^). Across the full experimental age range (c.a. 11.5 months) this translated into a 4.65-fold difference in the odds of feeding. The negative effect of prior infestation was larger than in the first experiment (*z* = −286.714, *P* < 0.001, Fig. 2). In this experiment, the odds of successfully feeding on previously infested mice were approximately one-quarter of those when feeding on naïve mice (e^βExposed^ = e^−1.439^ = 0.237). There was no evidence of an interaction between mouse age and prior exposure (*z* = −1.269, *P* = 0.204). As in the first experiments, the magnitude of the mouse-to-mouse variation was similar to that of the fixed effects. The standard deviations of the intercept and slope were 0.843 and 0.885, respectively.

### Feeding success in multiple experiments—post-hoc analysis

**Fig. 3:**
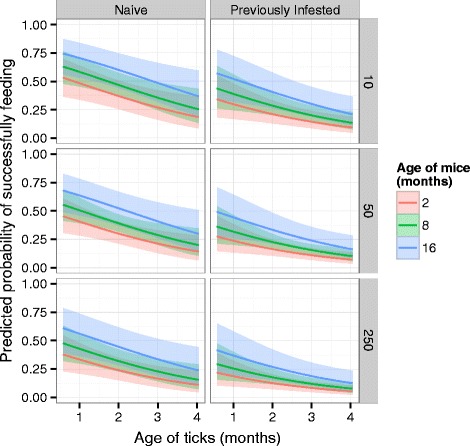
Combined dataset. The predicted probability of successfully feeding as ticks increase in age (x-axis) and density (rows), on naïve (left) or previously infested (right) hosts of increasing age (color scale) from the full model fit to the data from all eight experiments. The 95 % confidence intervals are for the fixed effects of the generalized linear mixed model without accounting for the random effects

Across 192 infestations (96 mice) an average of 29.2 % of RMWT larvae successfully fed. Consistent with the experiments presented above, feeding success declined with an increase in tick density, (*z* = −2.102, *P* = 0.036, Fig. 3) and with prior infestation (*z* = −5.354, *P* = <0.001, Fig. 3). Feeding success increased with age of the mouse (*z* = 2.125, *P* = 0.034, Fig. 3). The effect sizes for tick density and host age in this combined dataset were somewhat smaller than in the previous experiments: β_Dose_ = −0.455 versus −0.667 and β_Mouse Age_ = 0.067 versus 0.134 in the combined versus individual datasets. The effect of prior infestation, however, was nearly as large in the combined dataset as in the first experiments (β_Exposure_ = −0.786 versus −0.796). As in experiment 1, the interaction between tick density and prior infestation was not significant (*z* = −1.301, *P* = 0.193). Similarly, there was no evidence of an interaction between mouse age and prior infestation (*z* = −0.053, *P* = 0.957).

Because of the large variation in ages of the tick larvae in these combined experiments (19 – 118 days), we also tested whether there was an effect of tick age on feeding success. With every month of age increase, the odds of successfully feeding declined 0.63-fold (= e^βTickAge^ = e^−0.465^). Over 3.3 months in tick age, the odds of feeding declined to less than a quarter of those of the youngest ticks (= e^βTickAge × 3.3months^ = e^−0.465 x 3.3^ = 0.216). There was no evidence of an interaction between tick age and prior infestation of the host (*z* = −0.596, *P* = 0.551).

The magnitudes of the random effects of mouse identity and experiment in this combined dataset were similar to those observed in the first 2 experiments. The standard deviation of the intercept and slope were 0.685 and 0.826, respectively. The standard deviation of the random effect of experiment was slightly larger (=0.41), but was still smaller than the mouse-to-mouse variation. Lastly, the sex of the host did not significantly alter feeding success when included as a fixed effect in the full model (*z* = −0.182, *P* = 0.855).

### Consistency in feeding success

**Fig. 4:**
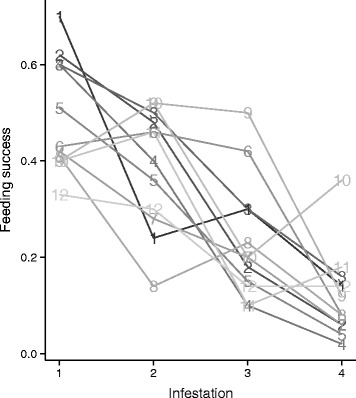
Consistency in feeding success. The proportion of ticks that successfully fed on each of 12 mice is shown when mice were exposed to ticks for the 1^st^, 2^nd^, 3^rd^ or 4^th^ time. The mice (lines) are labeled by the ranked feeding success from the 1^st^ infestation. The ranking of feeding success was not consistent across infestations, indicating no effect of mouse identity

In a single experiment mice were infested with ticks on four separate occasions. We used these mice to explore if the large mouse-to-mouse variation we observed in the three datasets was consistent over time. We fit a logistic model to the feeding success data on these mice (*n* = 12) with the main effects of density, mouse age, tick age and infestation number, but ignoring the effects of mouse identity. We then tested whether the residuals from this model (the variation left unexplained by the main effects) were attributable to mouse identity using an ANOVA (the residuals on the deviance scale were approximately normally distributed). There was no evidence that this residual variation in feeding success could be explained by mouse identity (*F* = 1.133, *P* = 0.366, Fig. 4). In other words, a mouse did not consistently feed a large or small number of ticks relative to other mice, once age and prior exposure were accounted for.

### Molting success

In all three datasets there was a statistically significant negative effect of mouse age on molting success of ticks that successfully engorged, although the effect sizes varied among datasets (Experiment 1: cohort 1 & 2: β_Mouse Age_ = −0.274, *z* = −2.305, *P* = 0.021; Experiment 2: β_Mouse Age_ = −0.16, *z* = −2.18, *P* = 0.029; Combined dataset: β_Mouse Age_ = −0.274, *z* = −2.207, *P* = 0.027). In the full, eight-experiment dataset the odds of successfully molting decreased 0.93-fold (= e^βMouseAge^ = e^−0.071^) with every month increase of mouse age. Because molting success was so high in this experiment—on average 94.7 % of fed ticks molted—the predicted probability of molting varied from a high of 0.98 with the youngest mice to a low of 0.94 with the oldest mice, all else being equal. In experiment 2 there was also a significant effect of prior infestation on molting success (β_Exposure_ = −0.638, *z* = −1.997, *P* = 0.046), but this effect was not observed in the first experiment or the combined dataset.

## Discussion

Blood-feeding is required for development and reproduction of many ectoparasitic arthropods and it is a key step in the transmission of most arthropod-borne pathogens. We hypothesized that prior infestation by ticks, the density of ticks, and ages of both the mouse and tick would alter blood-feeding success on deer mice. We observed that prior infestation of the host and increasing densities of ticks had negative effects on the feeding success of larvae (Fig. 1). Feeding success increased with host age, but decreased with tick age (Figs. 2 and 3). The majority of ticks that were able to feed successfully also molted successfully, but molting success declined slightly with host age. Finally, there was wide variation in tick feeding success from mouse-to-mouse across all experiments, suggesting a large stochastic effect on feeding, irrespective of host or tick properties.

Host defense, mediated through immunological responses and grooming behavior is a critical factor in host-ectoparasite interaction [10, 21, 22, 45]. These defenses can kill or remove ectoparasites. For example, after impala were exposed to a known density of host-seeking ticks, it was observed that the hosts restrained from grooming had nine times as many ticks compared to impala that were able to groom naturally [46]. Host defenses can also impair feeding success. For example, Hawlena *et al.* found that grooming decreased the amount of blood ingested by the flea *Xenopsylla conformis* when allowed to feed on rodents that were not restrained from grooming [47]. Although these defenses can be innate, they are often stimulated by repeated infestations and can change feeding success of the ectoparasite over time [8, 40, 47, 48]. For example, prior infestation of the bank vole with larval *I. trianguliceps* ticks stimulated host responses that reduced the engorgement weights of the larvae by 80 – 95 % [48]. In our study we did not measure defensive responses of host mice. Thus, it is possible that prior infestation caused non-defensive physiological changes (e.g., anemia) in the hosts that reduced blood-feeding. However, given the broad evidence for induced host defenses in animal-ectoparasite systems, it seems likely that immune responses and grooming produced the changes in feeding success on previously infested hosts. Regardless, our results clearly indicate that prior infestations of hosts will change the probability of successful feeding for subsequent RMWT larvae. As a result, the infestation histories among hosts in a population may shape the proportion of ticks that can feed and develop to the next life stage.

Density is another important variable in host-ectoparasite interaction. There have been conflicting observations of how ectoparasite density affects feeding success. Co-feeding can facilitate access to blood. This has been observed with sandflies, whereby groups of flies feeding together ingest more blood per capita than individual flies [49]. Similarly, Davidar *et al.* observed that *I. scapularis* ticks had greater engorgement at higher densities on white-footed mice (*Peromyscus leucopus*) [15]. Others have observed no effect of ectoparasite density on feeding success [32]. Alternatively, ectoparasites may compete for feeding sites on the host, causing a reduction in feeding success at higher densities. For example, Levin and Fish observed a negative effect of density on feeding success of *I. scapularis* larvae on white-footed mice [31]. We also observed a negative relationship between the density of RMWT larvae and feeding success. For every 10-fold increase in tick density the odds of successfully feeding decreased by approximately half. This suggests that feeding success of larval RMWTs may be partly regulated by tick density on a host. Estimates of deer mouse densities range from 5 to 15.4 ha^−1^ in the spring and 1 – 17.6 ha^−1^ in the fall [50]. This variation could impact tick burdens, because tick-host contact could change as the mouse population increases or decreases [17]. If mouse densities in the field were high it could distribute the tick population across a larger number of hosts (fewer ticks per host), which would increase feeding success. Conversely, when mouse densities decrease (e.g., predation) the tick population could become aggregated on a few individuals, decreasing blood-feeding success. These types of processes may contribute to the variability in ticks observed in the field from year-to-year [6].

Hypothetically, more ticks in the environment will result in higher burdens on hosts that contact those ticks [17, 33]. However, the process of tick-host contact plays out over time. This introduces the potential effect of age on ectoparasite feeding success. In multiple systems there is evidence that host age affects ectoparasites [12–14, 51–53]. For example, the abundance of the flea *Synosternus cleopatrae* was found to be lower on juvenile gerbils compared to adults [52]. Kiffner *et al.* found greater burdens of *Ixodes sp*. larvae on adult yellow-necked mice compared to sub-adults of the same species [13]. Similarly, Brunner and Ostfeld found *I. scapularis* larvae to be less abundant on juvenile white-footed mice (*P. leucopus*) compared to both sub-adult and adult mice [12]. In these studies it is unclear if the differences in burden reflect variation in tick contact (e.g., foraging behavior) among different aged hosts, or if the effects result from age effects after contact. Monello and Gompper observed higher numbers of fed *D. variabilis* ticks on older raccoons, indicating a biotic (host) effect after contact with ticks [53]. In our study the host mice were exposed to host-seeking ticks in a small space that reduced differences in host-tick contact. Thus, our results likely represent effects of host age on post-contact factors such as physiological condition, grooming behavior, or immunological defense. Interestingly, the observed increase in feeding success on older mice aligns with the reported studies of tick burdens in the field [12, 13, 52]. Thus, the age demographics of the host population may shape the probability of tick contact and successful blood-feeding.

As with hosts, ectoparasites may age physiologically between blood meals. Given that host contact is not predictable, ticks may remain in the environment for variable periods of time at each life stage before blood-feeding. Hazler and Ostfeld observed a decline in feeding success of *I. scapularis* larvae when fed on tick-naïve white-footed mice over a 2-month period [32]. In our study the change in RMWT feeding success was relatively small over the 1-month post-hatch period (Fig. 3). This suggests a larva that is able to attach within a month of hatching would not experience significant changes in feeding success. Beyond that period the tick feeding success declined, but it remains unclear if RMWT larvae survive that long in the field. Owen *et al.* observed that survival of RMWT larvae changed with ambient temperature and relative humidity, with most larvae surviving fewer than 10 days under conditions simulating natural variation [54]. Using radiolabeled RMWT larvae Sonenshine *et al.* estimated tick survival in field enclosures and by release-recapture [55]. Approximately 70 % of larvae survived up to 40 days in canisters covered with soil, rocks and litter [55]. However, very few released larvae were recovered from trapped hosts, and 90 % of the on-host recoveries occurred within 2 weeks of release. This suggests that most unprotected larvae did not survive beyond 14 days, or were unable to attach after that period [55]. Field studies of *D. variabilis* in the eastern United States reveal that habitat (e.g., vegetation) strongly affects the longevity of larval ticks off-host and underscore the idea that larvae may have a short period of time to find a host, feed and develop to the nymph stage [56].

Although our study was conducted in artificial conditions with nearly complete removal of variation in tick-host contact, the data reveal important details that should be considered in field studies. First, host age demographics and history of tick exposure are likely to change over a season and create dynamic conditions for tick feeding and development. For example, there are more adult mice in the early spring months and more juveniles during the later spring and early summer months [57]. Given our observation that larvae feed more successfully on older mice, it may be that ticks feeding early in the spring have an advantage over those that feed later when the host population is dominated by younger mice. However, it may also be that older mice have a higher probability of prior exposure to ticks and have acquired resistance that impairs blood-feeding. In that scenario, younger hosts may represent a better resource for the ticks because they have had no previous exposure to ticks. While blood-feeding on a naïve mouse would be more advantageous for the tick compared to a previously exposed mouse, our data suggest an older, resistant mouse would feed approximately the same number of ticks as a younger, naïve mouse (Fig. 2). Host age demographics and tick feeding success appear to interact in the field [53], but it remains to be determined how changes in the host population over time affect actual tick population dynamics. Another vital detail was the large stochastic effect on feeding success across all mice. Despite controlled conditions, there was wide variation in feeding success that could not be explained by host or tick traits. This was particularly clear with the subset of mice infested four times. Unlike field studies that suggest some mice consistently host high tick burdens [33, 34] our experiment indicated that mice do not intrinsically feed higher (or lower) numbers of larvae when tick contact, host age and the history of exposure are all controlled (Fig. 4). To accurately predict distributions of ticks, it may be necessary to determine how the mechanisms of tick-host contact interact with the factors that influence feeding success [33]. Finally, it should be noted that although molting success declined significantly with mouse age (98 % with the youngest mice to 94 % with the oldest mice), the majority of ticks that successfully fed also successfully molted. The approximate 4 % difference in development to the nymph stage is a small effect compared to the approximate 50 % reduction in feeding success when feeding on a previously infested host.

Pathogen transmission by vectors varies from year-to-year for many different reasons (e.g., vector density, host density, pathogen prevalence). As vector density increases, the probability of a transmission event and the risk of infection also increase [1]. These interactions have been observed in studies of Lyme disease. The development of Lyme disease involves the pathogen *Borrelia burgdorferi* sensu lato, which is transmitted by ticks in the genus *Ixodes* [58]. The dynamics of Lyme disease have been linked to forest fragmentation, tree masting, vertebrate community structure, and predator–prey dynamics [1, 58, 59]. However, the central driver of the disease is the interaction between *Ixodes sp*. and rodent hosts (e.g., white-footed mice; *P. leucopus*), which are the primary reservoirs for the pathogen [1]. When the population of mice increases, the population of ticks subsequently increases, which is expected to drive up the probability of pathogen transmission between ticks and mice [60]. However, if host status is important to determining the number of ticks in a population—as our data suggest—the likelihood of a transmission event may also vary with intrinsic properties of the ticks and mice. It remains to be determined how these intrinsic factors vary in the field populations of ticks and mice. Future research will shed light on the strengths of these effects in natural populations and will provide a more complete picture of how host-vector-pathogen dynamics play out over space and time.

## Conclusion

Our data reveal that prior infestation of the host, tick density, host age and tick age will each affect the blood-feeding success of larval Rocky Mountain wood tick larvae. These data suggest the biotic factors driving feeding success of RMWT larvae in the field could be (i) the ratio of naïve versus tick-exposed hosts in a population, (ii) the density of ticks in the environment, (iii) the ratio of young versus old hosts and (iv) the ages of the larvae at first encounter with a host. Small numbers of larvae that get on an older, naïve host earlier in the season will have a better chance of developing to the next life-stage. Variation in these factors may cause changes in tick population dynamics and the probability of pathogen transmission. These data add to the much needed information on biology and ecology of tick larvae, which will improve modeling of tick population dynamics.

## Additional files

Additional file 1:
**Post-hoc dataset – This additional file provides the feeding success data of RMWT larvae for the six additional experiments conducted and used in the post-hoc data analysis.**
Additional file 2:
**Consistency in feeding success – This additional file provides the feeding success data of RMWT larvae across four infestations on the 12 mice used in this experiment.**

## Abbreviation

RMWT: Rocky Mountain wood tick
